# The protective effects of different compatibility proportions of the couplet medicines for Astragali Radix and *Angelica sinensis* Radix on myocardial infarction injury

**DOI:** 10.1080/13880209.2020.1725581

**Published:** 2020-07-01

**Authors:** Lu Chen, Min Song, Lusha Zhang, Chunxiao Li, Zhirui Fang, Joel Wake Coffie, Liyuan Zhang, Lulu Ma, Leyu Fang, Qianyi Wang, Wenjie Yang, Fanggang Li, Xiumei Gao, Hong Wang

**Affiliations:** a Tianjin State Key Laboratory of Modern Chinese Medicine, Tianjin, China; b Key Laboratory of Pharmacology of Traditional Chinese Medical Formulae, Ministry of Education, Tianjin University of Traditional Chinese Medicine, Tianjin, China; c Tianjin Key Laboratory of Chinese Medicine Pharmacology, Tianjin University of Traditional Chinese Medicine, Tianjin, China; d Institute of Traditional Chinese Medicine, Tianjin University of Traditional Chinese Medicine, Tianjin, China; e Shandong Danhong Pharmaceutical Co., Ltd., Heze, China; f School of Integrative Medicine, Tianjin University of Traditional Chinese Medicine, Tianjin, China

**Keywords:** Apoptosis, inflammation, angiogenesis

## Abstract

**Context:**

Astragali Radix (AR) and *Angelica sinensis* Radix (ASR) combinations are used to treat cardiovascular disorders.

**Objectives:**

This study investigates the protective effects of different compatibility proportions of AR and ASR on cardiac dysfunction in a C57BL/6 mouse model of myocardial infarction (MI).

**Materials and methods:**

MI mice were induced by ligation of the left coronary artery and divided into six groups: sham, vehicle, 10 mg/kg/d simvastatin and combinations of AR and ASR at different ratios, including 1:1 (AR 2.5 g/kg + ASR 2.5 g/kg), 3:1 (AR 3.75 g/kg + ASR 1.25 g/kg) and 5:1 (AR 4.17 g/kg + ASR 0.83 g/kg). Both AR–ASR combinations and simvastatin were dissolved in saline solution and given daily by gavage. The left ventricle function, infarct size, heart tissue injury, apoptosis of cardiomyocytes, leukocyte infiltrates, capillary density and expression of cleaved caspase-3, cleaved caspase-9, Bcl-2, Bax, Bad, IL-1β, IL-6, VEGF, p-Akt and p-eNOS were analysed.

**Results:**

Different combinations of AR and ASR improve cardiac function and reduce infarct size (61.15% vs. 39.3%, 42.65% and 45.5%) and tissue injury through different mechanisms. When AR was combined with ASR at ratio of 1:1, the inflammation and cardiomyocyte apoptosis were suppressed (*p* < 0.05, *p* < 0.01). The combination ratio of 3:1 exerted effect in promoting angiogensis (*p* < 0.05). In the combination of AR and ASR at 5:1 ratio, angiogenesis was significantly improved (*p* < 0.01) and the apoptosis was inhibited (*p* < 0.05).

**Conclusions:**

Our results reflect the regulation of multiple targets and links in herb pairs and provide an important basis for the use of AR and ASR combinations in the treatment of MI.

## Introduction

Myocardial infarction (MI) and consequent heart failure are the most common types of cardiovascular disease and the leading causes of morbidity and mortality worldwide (Ibanez et al. 2018; Roth et al. 2018). MI develops when cholesterol particles accumulate on the walls of arteries due to various risk factors. The narrowed coronary arteries lead to decreased blood flow and eventually reduce the amount of oxygen supplied to heart muscle. The lack of oxygen can produce chest pain, angina pectoris or even a heart attack, which will impose a significant burden on quality of life (Heusch and Gersh 2017). The most effective treatment is to restore blood perfusion to relieve myocardial tissue hypoxia (Bagai et al. 2014). Current pharmacological treatments, such as inhibitors of the renin–angiotensin–aldosterone system (Kim et al. 2019) and β-adrenoreceptor blockers (Ibanez et al. 2013), prevent the expansion of myocardial necrosis to some extent; however, contraindications and adverse effects have limited their clinical application. To improve the quality of life and prognosis of patients with MI, it is critical to develop effective medicinal therapies which are both convenient to perform and well accepted. Moreover, as MI is a multifactorial disease with multiple mechanisms, as well as affecting various cell types, it is preferred to use the combination of additive or synergistic multitarget therapies for the optimal cardioprotection (Davidson et al. 2019).

Traditional Chinese medicine (TCM) herbs are widely used as medicines and daily dietary supplements in Asia on the mode action of ‘multi-component, multi-target, multi-pathway’ (Liu Z et al. 2016). According to Chinese medicine theory, promotion and activating blood circulation to remove blood stasis is one of the main treatment strategies in ameliorating MI. Astragali Radix (AR), the dried radix of *Astragalus membranaceus* (Fisch.) Bge (Fabaceae) known as Huangqi in Chinese, is a major medicinal herb considered to enrich vital energy and treat stagnant blood flow. *Angelica sinensis* Radix (ASR), the dried radix of *Angelica sinensis* (Oliv.) Diels (Apiaceae) known as Danggui in Chinese, has been used as a tonic agent to promote blood circulation for several years (Wei et al. 2016). To obtain synergistic effects and diminish possible adverse reactions, Chinese herbs are often used in formula. Herb pair is a centralized representative of herb compatibility. The AR–ASR combinations, especially the ‘Dang-Gui Decoction for Enriching the Blood’ with a combination of AR and ASR at a 5:1 ratio, have been used to invigorate ‘Qi’ and nourish blood for nearly 1000 years. Modern pharmacological studies demonstrated that combinations of AR and ASR have synergistic effect in stimulating red blood cell production and enhancing cardiovascular function (Lei, Gao, et al. 2003; Lei, Wang, et al. 2003; Chiu et al. 2007; Li et al. 2011; Ma et al. 2017; Li et al. 2017; Yan et al. 2018). The couplet medicines for AR and ASR may be considered as a complementary therapeutic medicine for MI. In the clinical application, besides the 5:1 ratio, other combination ratios of AR and ASR are used which vary from 1:5 to 5:1. In this study, we explored the effects of different compatibility proportions of AR and ASR in mice MI model to identify a reasonable combination for improving cardiac function.

## Materials and methods

### Reagents

The reference compounds of calycosin, formononetin, astragaloside IV (AS-IV), ferulic acid and ligustilide were purchased from Chinese National Institute for Control of Pharmaceutical and Biological Products (Beijing, China). Avertin and simvastatin were purchased from Sigma-Aldrich (St. Louis, MO). The anti-CD45, anti-CD31, α-tubulin, VEGF, p-eNOS, eNOS and biotin-conjugated goat anti-rabbit IgG polyclonal antibodies were obtained from Abcam (Cambridge, UK). Caspase-3, cleaved caspase-9, Bcl-2, Bax, Bad, p-Akt, Akt and GAPDH antibodies were bought from Cell Signalling Technology (Boston, MA). The *in situ* cell death detection kit and protease and phosphatase inhibitor cocktails were purchased from Roche Diagnostics (Mannheim, Germany). Interleukin 6 (IL-6) and interleukin 1 beta (IL-1β) Mouse ELISA kits were obtained from R&D Systems (Minneapolis, MN). Alex Fluor 594 Goat Anti-Rabbit IgG (H + L) was from Life Technologies (Waltham, MA).

### Preparation of Chinese herbal decoction

All crude herbs were purchased from the specialized market of Chinese herbal medicines (Anguo Traditional Chinese Medicine Market) in Anguo, China on 21 March 2017 to 23 March 2017. The two herbs were identified by Dr. Li Tianxiang (Experiment Teaching Department, Tianjin University of Traditional Chinese Medicine). Each voucher specimen was deposited at the Academy of Traditional Chinese Medicine of Tianjin University of Traditional Chinese Medicine. The voucher specimen numbers of AR and ASR are no. 20170527 and no. 20170516, respectively.

AR, ASR and different ratio combinations of AR–ASR (1:1, 3:1 and 5:1) were prepared. According to different formulations, the appropriate amounts of crude herbs were weighed separately to form a combined weight of 360 g (300 g AR and 60 g ASR for the 5:1 formulation, 270 g AR and 90 g ASR for the 3:1 formulation, 180 g AR and 180 g ASR for the 1:1 formulation). Before decocting, the medicinal materials were soaked in pure water for 1 h. The mixture was boiled in six volumes of water (v/w) (2160 mL) for 40 min the first time and four volumes of water (1440 mL) for the next two times. All the filtrates were mixed together and condensed to a total volume of 360 mL using a rotary vacuum centrifuge. The final concentration of AR, ASR and the combinations were 1 g/mL. The concentrate was sub-packaged and stored at –4 °C.

**Figure 1.**
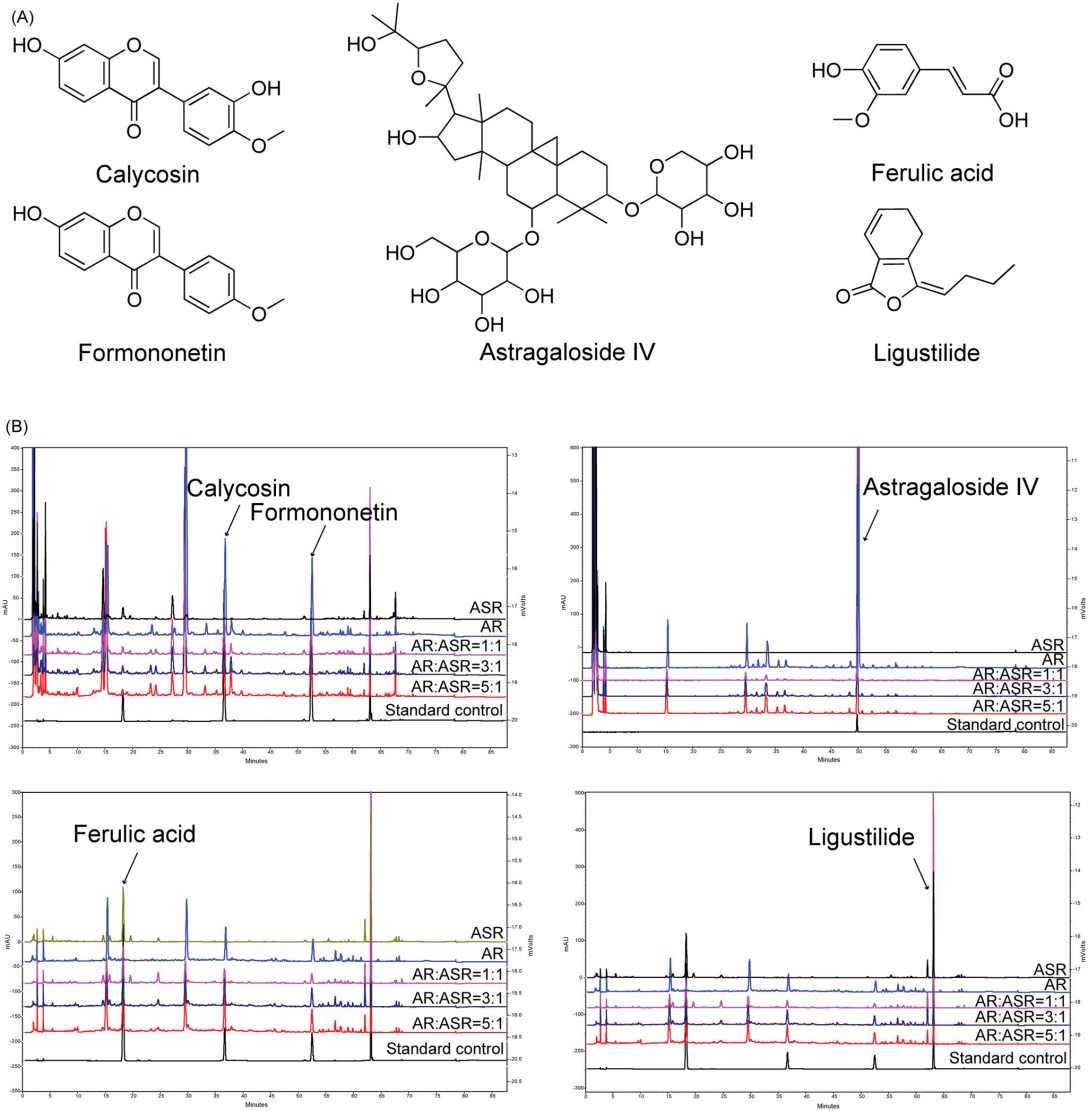
HPLC fingerprints of five components of AR and ASR. (A) The chemical structures of five characteristic compounds in AR and ASR. (B) HPLC fingerprints of AR, ASR and different combinations of AR and ASR. The results showed that the main components in AR and ASR, including calycosin at 260 nm, formononetin at 260 nm, astragaloside IV at 203 nm, ferulic acid at 316 nm and ligustilide at 320 nm, could be observed in each group.

Calycosin, formononetin and AS-IV act as effective compounds in AR (Shi et al. 2014). Ferulic acid and ligustilide are the characteristic components of ASR (Shi et al. 2014). The chemical structures of these five compounds are shown in Figure 1(A). The components were measured to identify AR, ASR and the combinations by high-performance liquid chromatography (HPLC). The final concentrations of the reference compounds of calycosin, formononetin, AS-IV, ferulic acid and ligustilide were 61.75, 59.50, 272, 6.25 and 118.75 μg/mL, respectively. HPLC was performed by an Agilent 1260 Infinity High Performance Liquid Chromatography (Agilent Technologies, Palo Alto, CA). Solutions (10 mL) of each group were precisely measured and extracted by water-saturated n-butanol for four times. The extract was dried and dissolved by methanol. After filtering through a 0.22 μm filter membrane, each filtered sample (10 μL) was separated on an Inertsil ODS-3 column (4.6 mm × 250 mm, 5 μm). Gradient elution was carried out with acetonitrile (A)–0.2% formic acid solution (B) as follows: 0–25 min, 15 → 25% A, 85 → 75% B; 25–50 min, 25 → 40% A, 75 → 60% B; 50–65 min, 40 → 90% A, 60 → 10% B; 65–75 min, 90 → 90% A, 10 → 10% B; 75–76 min, 90 → 15% A, 10 → 85% B; 76–88 min, 15 → 15% A, 85 → 85% B. The flow rate was 1.0 mL/min and the column temperature was 35 °C. Typical HPLC fingerprints of the five main components in AR, ASR and different combinations of AR and ASR are shown in Figure 1(B).

### Animals

Due to the presumption that cyclic hormonal changes of female mice across the ovulatory cycle introduce excess variability to measures of interest, male C57BL/6 mice of specific pathogen free (SPF), 22–25 g, were purchased from Beijing Weitong Lihua Experimental Animal Technology Co. Ltd. (Beijing, China) and maintained at the Animal Center of Institute of Biomedical Engineering, Chinese Academy of Medical Sciences (Tianjin, China). All animals were kept under 22–25 °C and a 12 h light/dark cycle with standard food pellets and free access to tap water. All animal care and experimental procedures were approved by the Animal Ethics Committee of Tianjin University of Traditional Chinese Medicine (TUTCM20170628) and performed in accordance with the approved guidelines on the use of laboratory animals. All experiments were performed in accordance with the Guide for the Care and Use of Laboratory Animals of the United States National Institutes of Health (Garber et al. 2011) and the Declaration of Helsinki.

### Myocardial infarction model and drug treatments

After one week of adaptation, 12-week old experimental mice underwent surgery to induce acute MI by ligation of the left anterior descending (LAD) coronary artery, as described before (Kolk et al. 2009; Reichert et al. 2017). In brief, after anaesthesia with injection of Avertin (0.33 mL/20 g) into the abdominal cavity, mice were orally intubated and artificially ventilated on a rodent respirator. Hearts were then exposed through the left lateral thoracotomy. The LAD coronary artery was visualized and ligated with an 8-0 suture line. The occlusion of coronary was confirmed by pallor and regional wall motion abnormality of the left ventricle (LV). Mice in the sham group underwent the same time-matched surgical procedure without ligation. All the operations were performed by an experienced surgeon who was blinded to the experimental groups.

Post-operatively, the mice were randomly divided into six groups: (1) sham group (*n* = 12). Mice had no ligation and they were orally administered with saline solution; (2) vehicle group (*n* = 15). The mice with surgery received saline solution orally; (3) simvastatin group (as a positive control drug) (*n* = 12). The mice were treated with 10 mg/kg/d simvastatin by gavage; 4–6. AR–ASR combination groups with different ratios (1:1, 3:1 and 5:1) (*n* = 15 for each group). Before drug treatments, the combinations were diluted with saline solution into 0.5 g/mL. The mice were administered with 10 mL/kg/d plant extracts based on the doses used in clinical practice. Both the AR–ASR combinations and simvastatin were dissolved in normal saline solution and given daily by gavage starting on day 0. Drugs were given for four consecutive weeks. At 7 and 28 days after MI surgery, mice were sacrificed. Hearts were perfused with cold phosphate buffered solution (PBS) and a portion of the LV was either frozen in liquid nitrogen for biochemical analysis or fixed in 4% paraformaldehyde for histological analysis.

### Echocardiography

Cardiac function was examined by transthoracic echocardiography with a Vevo 2100 high-resolution ultrasound biomicroscope (VisualSonics, Toronto, Canada) and Vevo analysis software at days 7, 14 and 28 post-MI. The investigator was blinded to group assignment. Mice were anaesthetized with 2% isoflurane inhalation and placed in supine position. Heart rates were maintained at 400–500 beats per minute for measurements. Images were obtained from the B-mode parasternal long axis view and M-mode of the parasternal short-axis view to calculate the cardiac diastolic and systolic functions. Left-ventricular end-systolic volumes (LVESVs) and left-ventricular end-diastolic volumes (LVEDVs) were measured through parasternal long-axis scans. The left-ventricular fractional shortening (LVFS) and left-ventricular ejection fraction (LVEF) were calculated in accordance with modified American Society of Echocardiography recommended guidelines.

### Histological and immunofluorescent assessments

Mice received drug once a day for 4 weeks after surgery. Hearts were harvested after the 28-day echocardiographic analysis. Heart tissue was fixed with 4% paraformaldehyde, dehydrated, embedded in paraffin, and transversely sectioned into 5 µm pieces. Masson’s trichrome staining was performed to evaluate the degree of fibrosis. Fibrotic tissue was stained to blue-grey and viable myocardium to red. The fibrosis area was calculated as the ratio of the length of fibrotic area to the length of left-ventricular inner circumference with Image J software. The haematoxylin and eosin (H&E) staining was performed to detect tissue recovery after ischaemia injury, as described previously (Baudouy et al. 2017).

For immunohistochemical labelling, paraffin sections were warmed to 60 °C for 5 min and then rehydrated through xylene and a series of graded ethanol solutions. Sections were incubated with preheated antigen retrieval buffer (0.1 M sodium citrate buffer, pH 6.0) for 10 min. After washing in PBS 3 times, blocking was done by 5% foetal bovine serum in PBS for 1 h at room temperature.

To assess post-MI leukocyte infiltrates, mouse hearts were immunostained for CD45 at seven days post-MI and CD45 positive cells were quantified in the peri-infarct zone. Anti-CD45 antibody (ab10558, Abcam, Cambridge, UK, diluted 1:100 with milk) was used for immunohistochemistry of leukocytes. Samples were incubated with primary antibody for 24 h at 4 °C. A biotin-conjugated goat anti-rabbit IgG polyclonal (ab205718, Abcam, Cambridge, UK, 1:500) was used as the secondary antibody.

Primary anti-CD31 antibody (ab28364, Abcam, Cambridge, UK, diluted 1:20 with 1% foetal bovine serum in PBS) was incubated with the sections overnight at 4 °C. Negative controls were without primary antibody. Secondary antibody (Alex Fluor 594 Goat Anti-Rabbit IgG (H + L), diluted 1:200 with 1% foetal bovine serum in PBS) was then incubated with the sections for 1 h in the dark at room temperature. Finally, the sections were viewed and photographed using a Nikon TI-U fluorescence microscope (Tokyo, Japan).

### Terminal deoxynucleotidyl transferase UTP nick end labelling (TUNEL) assay

For this parameter, mice received drug once a day for 4 weeks after surgery and were sacrificed 28 days after surgery. Apoptosis of cardiomyocytes was detected by TUNEL assay which was performed on paraffin-embedded sections using the *in situ* cell death detection kit at 28 days after treatment with different combinations of AR and ASR. According to manufacturer’s instructions, the dewaxed and rehydrated slides were incubated with 0.1% Triton X-100 for 8 min, rinsed twice with PBS, and then incubated in TUNEL reaction mixture for 60 min at 37 °C. After washed with PBS, samples were analysed by fluorescence microscopy in the range of 520–560 nm and detected in 570–620 nm. The number of TUNEL-positive nuclei was counted at ×200 magnification, 10 fields per section blinded to the study group. TUNEL^+^ cell density (%) is expressed as the ratio of TUNEL-positive nuclei to the total number of nuclei.

### Western blotting analysis

Western blotting analyses were performed to detect the cleaved caspase-3, cleaved caspase-9, B-cell lymphoma 2 (Bcl-2), Bcl-2-associated X protein (Bax), Bcl-2-associated death promoter (Bad), p-Akt, Akt, p-eNOS, eNOS, vascular endothelial growth factor (VEGF) protein expressions at day 28 after surgery. The heart tissues were homogenized in RIPA lysis buffer (50 mM Tris–HCl, pH 7.4, 150 mM NaCl, 1% Triton X-100, 0.1% SDS, 1% sodium deoxycholate, 1 mM sodium vanadate, 1 mM PMSF, 10 μg/mL aprotinin, 10 μg/mL leupeptin, 10 mM sodium fluoride) with a cocktail of protease and phosphatase inhibitors using tissue homogenizers. The mixture was spun 5000×*g* for 10 min to pellet unresolved fragments. Supernatants were collected and the protein concentration was measured by BCA method. After diluting in sample buffer (62.5 mM Tris–HCl, pH 6.8, 10% (v/v) glycerol, 2% SDS, 0.1% bromophenol blue), the protein samples were separated by 10% sodium dodecylsulphate polyacrylamide gel electrophoresis (SDS-PAGE) and transferred to PVDF membranes. Membranes were blocked with blocking buffer (5% skim milk or 5% BSA in TBS with 0.1% Tween 20) for 1 h. Membranes were then incubated with primary antibodies at 4 °C overnight. Twelve primary antibodies were used in the experiments: caspase-3 (9662, Cell Signalling Technology, Boston, MA), cleaved caspase-9 (9509, Cell Signalling Technology, Boston, MA), Bcl-2 (3498, Cell Signalling Technology, Boston, MA), Bax (2772, Cell Signalling Technology, Boston, MA), Bad (9268, Cell Signalling Technology, Boston, MA), α-tubulin (ab52866, Abcam, Cambridge, UK), VEGF (ab51745, Abcam, Cambridge, UK), p-Akt (4060S, Cell Signalling Technology, Boston, MA), Akt (4691S, Cell Signalling Technology, Boston, MA), p-eNOS (ab76199, Abcam, Cambridge, UK), eNOS (ab66127, Abcam, Cambridge, UK) and GAPDH (2118S, Cell Signalling Technology, Boston, MA). After washing, appropriate secondary antibodies were applied to the membranes for 1 h at room temperature. The reactive bands were developed using chemiluminescence according to the manufacturer’s instruction. Images were scanned and band intensities were analysed with photoshop CS5 software (Adobe Systems, San Jose, CA).

### Enzyme-linked immunosorbent assay (ELISA) analysis

Levels of IL-1β and IL-6 in heart tissues were detected by mouse ELISA kits. Heart tissues were harvested at seven days after surgery and were frozen in liquid nitrogen. The extracted tissues were homogenized in cold PBS, containing EDTA-free protease inhibitor, phosphatase inhibitor and PMSF mixture. The concentrations of IL-1β and IL-6 in the myocardium were determined by ELISA according to the manufacturer’s instructions.

### Statistical analysis

Statistical analysis was performed using SPSS software (version 17.0, SPSS, Chicago, IL). Data were expressed as mean ± standard deviation (S.D.). Differences between two groups were estimated with Student’s *t*-test. The results were analysed by one-way analysis of variance (ANOVA) for comparison of each parameter among two or more independent groups. Values of *p* < 0.05 were considered statistically significant.

## Results

### Different combinations of AR and ASR improve cardiac function and reduce infarct size after MI

**Figure 2.**
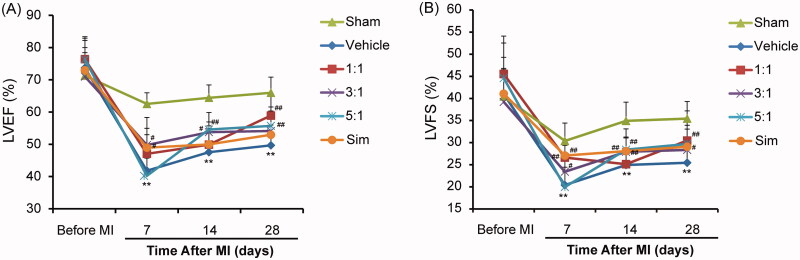
Treatments of different ratios of AR and ASR improve cardiac function after MI. (A, B) Echocardiographic measurements were performed on days 0, 7, 14 and 28 to detect the cardiac protective effects of different compatibility proportion of the couplet medicines for AR and ASR. Different ratios of AR and ASR improved cardiac function following MI in terms of LVEF (A) and LVFS (B) after post-infarction. *n* = 12 in each group. Scale bar, 1 mm. *n* = 6 in each group. The data are expressed as mean ± S.D. **p < 0.01 vs. sham mice treated with saline; ^#^*p* < 0.05, ^##^*p* < 0.01 vs. vehicle mice treated with saline. Sim: simvastatin.

To test the therapeutic effects of different combinations of AR and ASR, a mouse model of acute MI was established via permanent ligation at the middle of the LAD coronary artery, as previously described (Reichert et al. 2017). Cardiac function was examined by performing echocardiographic measurements of LVEF and LVFS at 0, 7, 14 and 28 days after MI. After that, mice were sacrificed, and infarct size and extent of fibrosis were evaluated by Masson’s trichrome staining of heart tissue sections. Echocardiographic results are shown in Figure 2. The results show that the combinations of AR and ASR at 1:1 ratio significantly elevated LVEF at 28 days (Figure 2(A)), and LVFS at 7 and 28 days (Figure 2(B)) post-MI (*p* < 0.01). AR–ASR mixtures of 3:1 significantly increased the LVEF at 7 and 14 days (Figure 2(A)), and LVFS at 14 days (Figure 2(B)) post-MI (*p* < 0.05 or *p* < 0.01). LVEF (Figure 2(A)) and LVFS (Figure 2(B)) were also significantly higher at 14 and 28 days after AR–ASR mixed at 5:1 treatment, compared with those of untreated MI hearts (*p* < 0.01).

**Figure 3.**
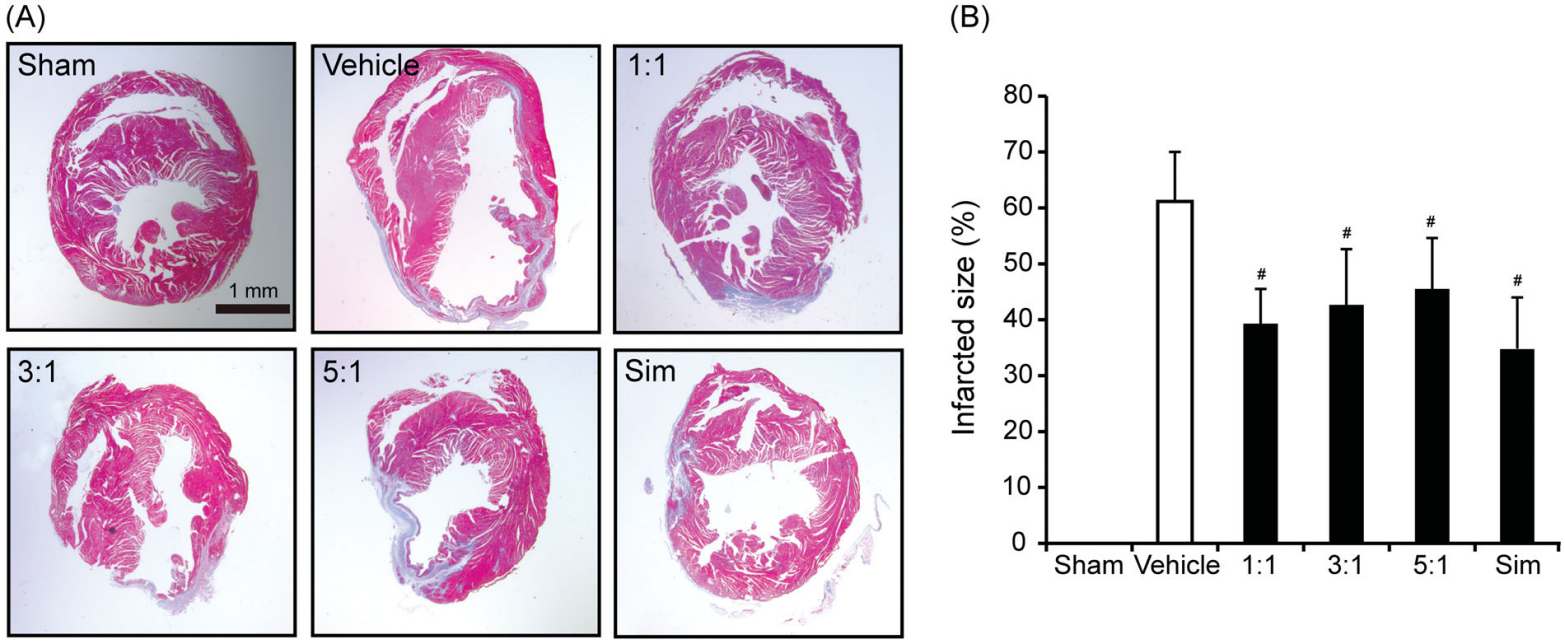
Treatments of different ratios of AR and ASR reduce infarct size after MI. (A) Infarct size was evaluated on Masson’s trichrome staining at 28 days after MI. The myocardial fibrotic tissue is defined on the basis of the difference in signal intensity between fibrotic tissue and viable myocardium and this difference generates the image contrast. (B) Quantitative analysis of fibrosis area at 28 days after MI. The fibrosis area was calculated as the ratio of the length of fibrotic area to the length of left-ventricular inner circumference with Image J software. Scale bar = 1 mm. *n* = 6 in each group. The data are expressed as mean ± S.D. ^#^*p* < 0.05 vs. vehicle mice treated with saline. Sim: simvastatin.

Infarct size is shown in Figure 3. Masson’s trichrome-stained myocardial sections at 28 days suggest the area of fibrosis was significantly reduced in different combinations of AR–ASR treated mice than that in vehicle mice on day 28 (*p* < 0.05) (Figure 3(A,B)). These results suggest that AR–ASR combinations at 1:1, 3:1 and 5:1 all improved cardiac performance by enhancing the preservation and/or recovery of functional myocardial tissues.

### Different combinations of AR and ASR reduce tissue injury in mice heart tissue

**Figure 4.**
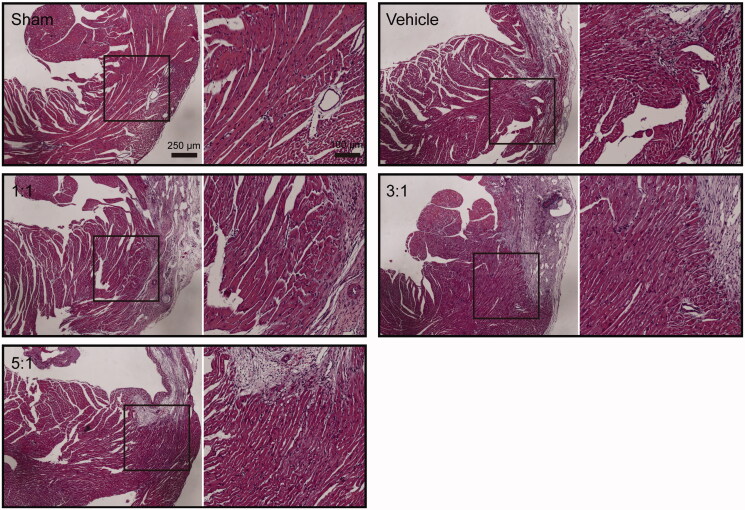
Treatments of different ratios of AR and ASR reduce border zone tissue injury in hearts of mice following MI. Haematoxylin–eosin staining of heart tissue confirmed the improvement by AR and ASR combination therapy. *n* = 6 in each group. Scale bar, 250 μm for the left sides, and 100 μm for the right sides of each figure.

The border zone tissue injuries in hearts of mice are shown in Figure 4. Haematoxylin–eosin staining was performed to observe the morphology and arrangement of cardiomyocytes in border zone of infarction. Histological assessment confirmed the improvement by different combinations of AR and ASR treatment when compared with that of vehicle (Figure 4). In the sham group, the morphology of the myocardial cells was uniform and the myofilaments were arranged neatly. In the model mice treated with saline, myocardial tissues were ruptured and curled with disorderly arranged myocardial fibres and infiltration of a large number of lymphocytes. However, in the AR–ASR combinations-treated groups, the myocardial fibres arranged orderly with less infiltration of inflammatory cells (Figure 4), indicating tissue injury in mice heart tissue was reduced in groups receiving combinations of AR–ASR at 1:1, 3:1 and 5:1 ratios.

### The combinations between AR and ASR at 1:1 and 5:1 inhibit apoptosis after MI

**Figure 5.**
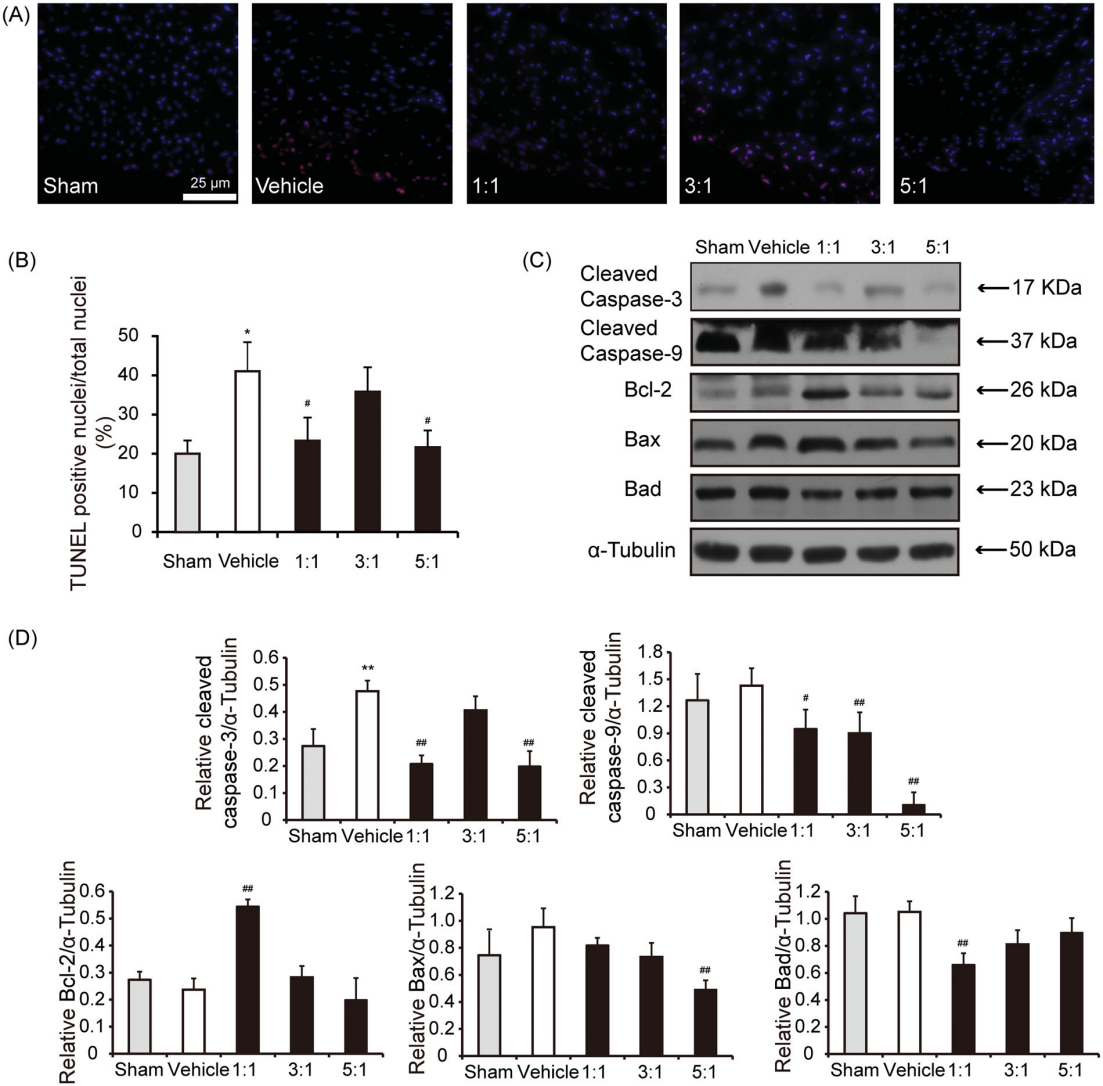
Treatments of AR–ASR combinations at 1:1 and 5:1 inhibit cardiomyocytes apoptosis. (A) TUNEL staining for detecting apoptosis of cardiomyocytes and DAPI for nuclear staining at day 28 after MI surgery and treatment with different combinations of AR and ASR or saline. Scale bar = 25 μm. (B) The percentage of TUNEL positive cells vs. the total DAPI-positive cells. Fewer TUNEL-positive cells were counted in the 1:1 and 5:1 AR–ASR combinations treated groups. *n* = 3 in each group. Values are expressed in mean ± S.D. *p < 0.05 vs. sham mice treated with saline, ^#^*p* < 0.05 vs. vehicle mice treated with saline. (C) Caspase-3 and caspase-9 activation (indicated by the expression of the active form of caspase, cleaved caspase-3), protein expression of Bcl-2, Bax and Bad were determined by Western blotting. α-Tubulin served as loading control. (D) Analysis of protein expression ratios of cleaved caspase-3, cleaved caspase-9, Bcl-2, Bax and Bad to α-Tubulin in infarct border zone on day 28 after surgery. *n* = 3 in each group. Values are expressed in mean ± S.D. **p < 0.01 vs. sham mice treated with saline, ^#^*p *< 0.05, ^##^*p* < 0.01 vs. vehicle mice treated with saline.

To determine the mechanisms responsible for the protective effects against cardiac dysfunction and remodelling of AR–ASR combinations, apoptosis, inflammation and angiogenesis were respectively assessed at 1 week or 4 weeks post MI. We first examined the cardiomyocytes apoptosis by TUNEL staining in the infarct border zone of mice treated with saline or different combinations of AR and ASR. Nuclei were stained blue with DAPI. As shown in Figure 5(A,B), there was little staining in the sham tissue. In vehicle group, the number of TUNEL-positive nuclei was significantly increased. Mice receiving combinations of AR–ASR at 1:1 and 5:1 had a lower number of apoptotic cells compared with vehicle treatment (*p* < 0.05). AR–ASR mixed at 3:1 had no significant effect on apoptosis after MI. Consistent with this, AR mixed with ASR at 1:1 ratio decreased activation of caspase-3 and caspase-9 (*p* < 0.05 or *p* < 0.01), increased Bcl-2 (protein promoting cellular survival and inhibiting the activities of pro-apoptotic proteins) expression (*p* < 0.01) and decreased Bad (pro-apoptotic protein) expression (*p* < 0.01) in response to MI. AR–ASR mixture at 5:1 ratio also decreased activation of caspase-3 and caspase-9 (*p* < 0.01), and downregulated Bax (pro-apoptotic protein) expression significantly (*p* < 0.01) (Figure 5(C,D)). These results suggested that the combinations of AR and ASR at 1:1 and 5:1 reduced tissue injury partially by inhibiting the apoptosis in ischaemic heart tissue.

### The combination between AR and ASR at 1:1 reduces inflammation after MI

**Figure 6.**
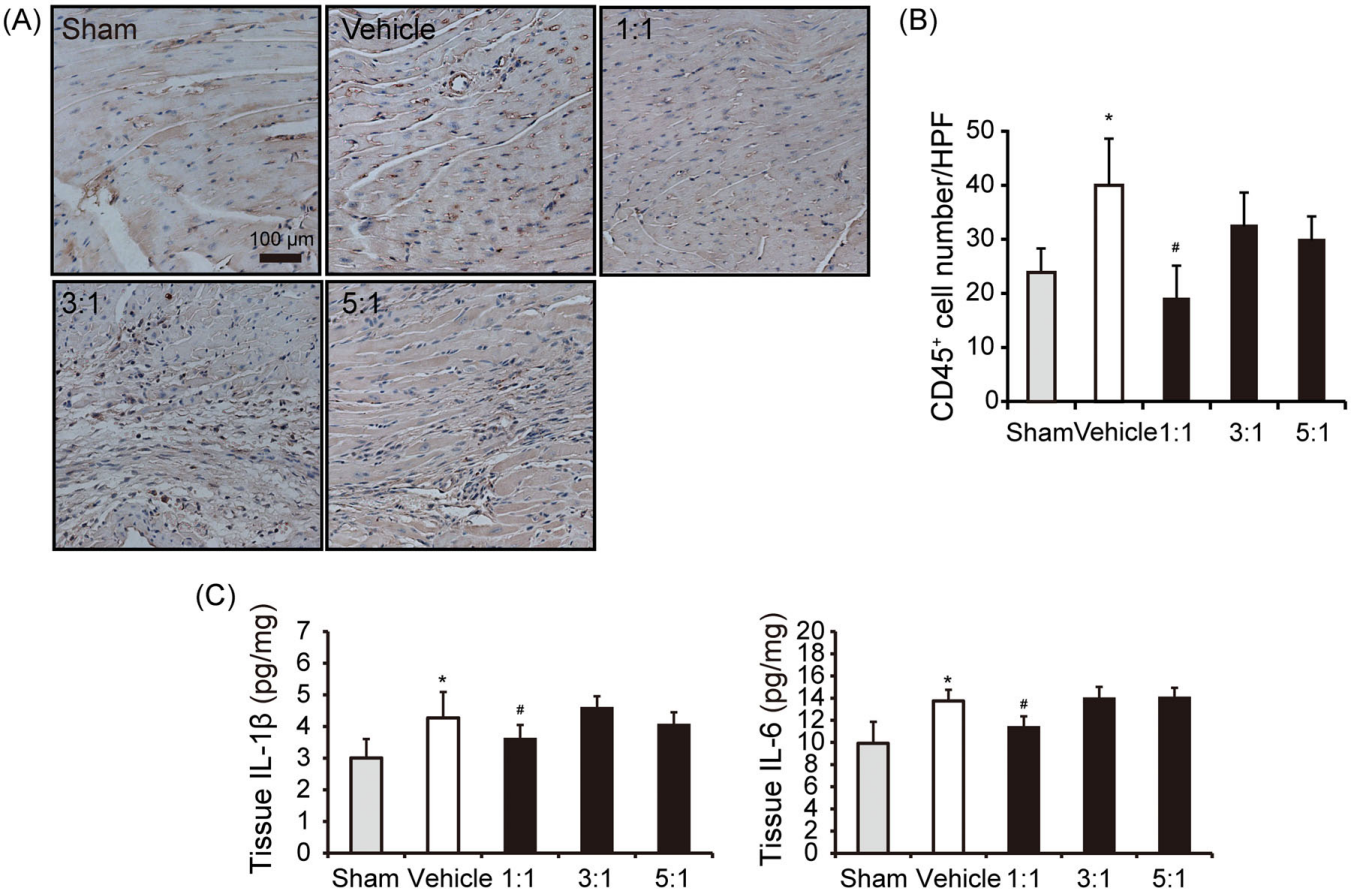
The combination between AR and ASR at 1:1 reduces inflammation after MI. (A, B) Myocardial white blood cells infiltrates. CD45^+^ staining on day 7 following LAD coronary ligation in hearts. Data are shown as mean CD45-positive count per high powered field. *n* = 3 in each group. Values are expressed in mean ± S.D. *p < 0.05 vs. sham mice treated with saline, ^#^*p* < 0.05 vs. vehicle mice treated with saline. (C) Changes of IL-1β and IL-6 levels in the infarct myocardium following coronary artery occlusion. Notably, a significant change of IL-1β and IL-6 content in the infarct myocardium occurred at 7 days post-MI. *n* = 6 in each group. Values are expressed in mean ± S.D. *p < 0.05 vs. sham mice treated with saline, ^#^*p* < 0.05 vs. vehicle mice treated with saline. IL-1β: interleukin 1 beta; IL-6: interleukin 6.

The infarct expansion will be promoted by recruitment and activation of white blood cells. To assess leukocyte infiltrates post-MI, heart tissues in the peri-infarct zone were immunostained for CD45 at seven days post-MI. The results showed that leukocyte infiltrates were significantly decreased in group receiving combination of AR–ASR at 1:1 ratio (*p* < 0.05) (Figure 6(A,B)). Moreover, ELISA was performed to detect the pro-inflammatory cytokines release in heart tissue. IL-1β and IL-6 were increased in mice after MI induced by LAD ligation (*p* < 0.05). Treatment of AR–ASR mixed at 1:1 significantly reduced the expression levels of IL-1β and IL-6 in the infarcted myocardium compared with those in the vehicle group (*p* < 0.05) (Figure 6(C)). These results suggested that the combination between AR and ASR at 1:1 exerted an anti-inflammatory effect in MI mice.

### The combinations between AR and ASR at 3:1 and 5:1 enhance angiogenesis after MI

**Figure 7.**
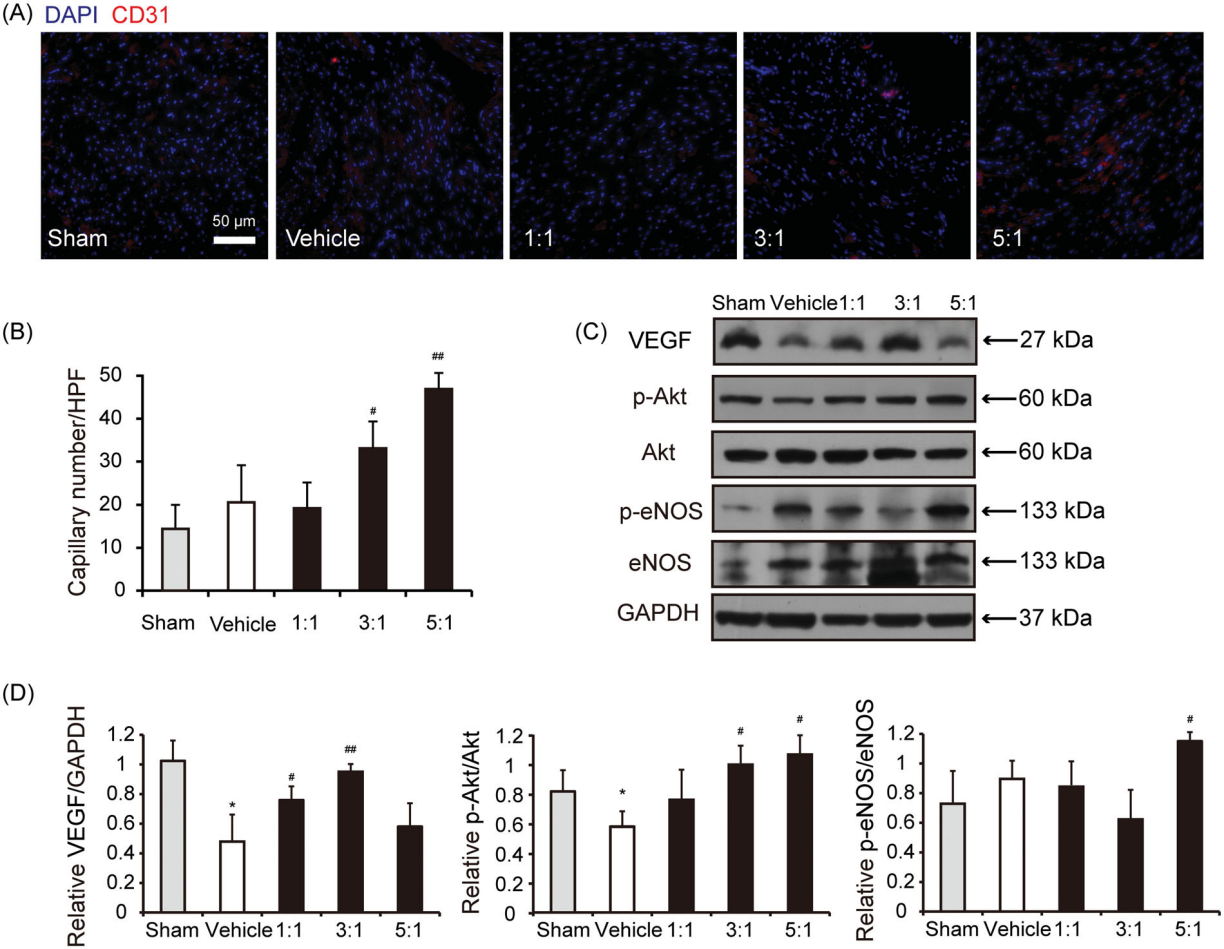
Combinations of AR and ASR at 3:1 and 5:1 increase border zone capillary density in hearts of mice at 28 days after MI. (A) Representative immunofluorescence images taken within the infarct border zone of mice treated with saline, or different combinations of AR and ASR. Capillaries were stained with CD31, nuclei were stained with DAPI. Scale bar = 50 μm. (B) Graph depicting the quantification of border zone capillary number across treatments presented as the number of CD31-positive capillaries per high-power field (HPF). *n* = 6 in each group. Values are expressed in mean ± S.D. ^#^*p* < 0.05, ^##^*p* < 0.01 vs. vehicle mice treated with saline. (C, D) Representative example and quantification of VEGF, p-Akt and p-eNOS protein levels (Western blotting) in the myocardial tissue at 28 days after MI surgery and treatment with different combinations of AR and ASR or saline. *n* = 4 in each group. Values are expressed in mean ± S.D. *p < 0.05 vs. sham mice treated with saline, ^#^*p* < 0.05, ^##^*p* < 0.01 vs. vehicle mice treated with saline.

To further detect the effect of different combinations of AR and ASR on MI-induced angiogenesis, the histological assessment was performed. At the border zone of the ischaemic region, capillary density was evaluated by identifying CD31^+^ vessels. Remarkably, the overall capillary density at the border zone of ischaemic myocardium 4 weeks after MI was significantly higher in the AR–ASR mixtures of 3:1 and 5:1-treated mice compared with the saline-treated animals (*p* < 0.05 or *p* < 0.01) (Figure 7(A,B)). These findings show that the combinations between AR and ASR at 3:1 and 5:1, especially the ratio of 5:1, increased neovascularization after MI.

To elucidate the molecular mechanism, western blot analyses were performed to detect the VEGF, p-Akt and p-eNOS protein expressions at day 28 after surgery. VEGF is an important cytokine involved in creating new blood vessels in hearts of patients with ischaemic heart disease (Crafts et al. 2015). Akt/eNOS pathway can regulate cell survival, migration, tube formation and NO release, which is essential for the repair of ischaemic tissue damage (Shiojima and Walsh 2002; Amano et al. 2003; Chen et al. 2005). As shown in Figure 7(C,D), treatment of combinations of AR and ASR at 3:1 significantly augmented VEGF protein expression (*p* < 0.01) and increased Akt phosphorylation (*p* < 0.05). AR mixed with ASR at 5:1 significantly increased Akt and eNOS phosphorylation (*p* < 0.05). The above results indicated that the acceleration of improved angiogenesis in AR–ASR combinations at 3:1 and 5:1-treated hindlimb ischaemic mice may be explained, at least in part, by the activation of pro-angiogenic factors.

## Discussion

In this study, the effects of different combinations of AR and ASR on cardiac protection were investigated in a mouse model of MI. The results suggested that different ratio combinations of AR and ASR improve cardiac function and reduce infarct size and tissue injury through different mechanisms. The combination at 1:1 inhibited apoptosis and reduced inflammation after MI. The combination ratio of 3:1 exerted effect in promoting angiogensis. The 5:1 ratio of AR–ASR not only reduced apoptosis, but also promoted angiogenesis post-MI significantly. Together, these observations demonstrated that different combinations of AR–ASR exerted protective effects against cardiac dysfunction. Although the mechanisms are diverse among different ratios, the herb pairs of AR and ASR could be exploited as a promising strategy for the prevention and treatment of MI.

Compatibility of AR and ASR is commonly utilized in Traditional Chinese Medicine to treat cardiovascular diseases. The most popular combination is ‘Dang-Gui Decoction for Enriching the Blood’, which is a traditional Chinese formulation comprising AR and ASR at a weight ratio of 5:1. It is widely used for promoting haematopoiesis as well as enhancing cardiovascular function. Modern pharmacological studies showed that the AR–ASR combinations can promote haematopoiesis (Li et al. 2017), stimulate NO production in endothelial cells (Gong et al. 2016), protect the cardiomyocytes from H_2_O_2_ injury through improving cell antioxidant ability (Li et al. 2011) and ameliorate coronary artery ligation-induced myocardial ischaemia associated with MAPK/NF-кB pathway (Ma et al. 2017). In the formula, AR is sweet and tepid and acts as the *Emperor* role. AR is considered to be the best herb to treat the ‘Qi’ (vital energy) deficiency and showed promising effects in improving biochemical and histological changes of heart failure (Yang et al. 2012; Gong et al. 2018). Astragalus injection made from AR has been widely used clinically for the treatment of heart disease (Ren et al. 2016). ASR is sweet and warm and acts as the *Minister* role in the combinations. It is widely used in clinic to invigorate blood circulation and regulate menstruation. Experimental and clinical studies have highlighted that ASR could reduce ischaemic injury and improve cardiac function after MI (Ma et al. 2015; Wei et al. 2016). The major bioactive constituents of AR and ASR show myocardioprotective effects against MI injury. For example, AS-IV from AR can significantly reduce the myocardial infarct size and increase shortening fraction (Zheng et al. 2018) by a variety of mechanisms including promoting angiogenesis (Yu et al. 2015), regulating energy metabolism (Tu et al. 2013), antioxidant (Zhang et al. 2006), anti-inflammation (Lu et al. 2015; Cheng et al. 2016) and anti-apoptosis (Si et al. 2014; Yin et al. 2019). *Astragalus* polysaccharides from AR improve cardiac function via ROS-p38 and PI3K/AKT signalling pathways and suppress cardiomyocyte apoptosis (Liu et al. 2018). *Angelica sinensis* polysaccharides from ASR can attenuate hypoxia-induced cardiomyocyte cell injury by down-regulation of miR-22 (Pan and Zhu 2018) and protect cardiomyocytes against oxidative injury and endoplasmic reticulum stress by activating ATF6 pathway (Niu et al. 2018). In the present study, we compared the effects of the compatibility of AR and ASR in different proportion on cardioprotection in a mouse MI model. AR–ASR combinations treatment significantly promoted cardiac function and reduced infarct size following a 28-day therapy, emerging as an effective approach in the treatment of MI patients.

The underlying mechanisms that different combinations of AR and ASR protect heart from coronary artery ligation-induced myocardial ischaemia are essential to be illustrated. The variation in the contents of bioactive components may account for the different effects of AR–ASR combinations at different ratios. Our preliminary studies (data not shown) demonstrated that AR-derived AS-IV, calycosin, formononetin, ASR-derived ferulic acid and total polysaccharides from AR and ASR are higher in combinations of AR–ASR at 5:1 ratios. However, ASR-derived ligustilide is higher in the 1:1 ratio. At 3:1 ratio, the contents of the above active substances are all at the intermediate level. This is consistent with previous reports on chemical assessment of the herbal decoction containing AR and ASR (Dong et al. 2006; Gao et al. 2007; Zheng et al. 2010; Zhang et al. 2012). The exact mechanisms underlying the differences remain to be elucidated by further studies.

In the clinical practice of herbal practitioners, the compatibility amongst all the herbs within a decoction will be revised according to the syndrome of the patients. Thus, the investigation of the underlying mechanisms on cardioprotective effects of different AR–ASR combinations is necessary. When AR was combined with ASR at the ratio of 1:1, leukocyte infiltrates were decreased and the inflammatory cytokine secretion was inhibited in the heart tissue compared with the vehicle group after seven-day treatment. At the meantime, the cardiomyocyte apoptosis was suppressed, partially through inhibiting activation of caspase-3 and caspase-9 and decreasing the ratio of Bad to Bcl-2. However, in the combination of AR and ASR at 5:1 ratio, the angiogenesis was significantly improved along with enhanced Akt-eNOS phosphorylation. After MI, a step-by-step myocardial remodelling takes place involving the inflammatory phase and the reparative phase. During inflammatory phase, the milieu in the heart is highly inflammatory. Sequential infiltration of the injured myocardium with neutrophils, monocytes and their descendant macrophages contribute to the initiation of inflammation (Soehnlein and Lindbom 2010; Mantovani et al. 2011; Liu et al. 2016). The excessive inflammatory response will result in the necrotic area being replaced with granulation tissue and eventually a collagen-rich scar (Prabhu and Frangogiannis 2016). It suggests that the combination of AR–ASR at 1:1 can be applied as an adjuvant therapeutic agent during the early stages post-MI to promote effective resolution of inflammation, and eventually facilitate cardiac repair and improve LV function. In the reparative or proliferative phase, neovascularization of viable myocardium in the infarct border zone is crucial during the process of tissue remodelling (Cochain et al. 2013). The efficient perfusion is required to prevent cardiomyocyte death, which can lead to infarct expansion, left ventricular dilation and heart failure (Shiojima et al. 2005). As the combination ratio of 5:1 exerted the strongest effects in promoting angiogenesis and inhibiting cardiomyocyte apoptosis, it can serve as a late-stage therapeutic agent for acute MI.

In this study, we found that treatments with different combinations of AR and ASR dramatically improved cardiac function, decreased myocardial infarct size and reduced tissue injury in MI mice. Moreover, the myocardial protective effects of AR combined with ASR at 1:1, 3:1 and 5:1 treatment were respectively achieved by inhibiting inflammation and apoptosis, promoting angiogenesis, and regulating angiogensis and apoptosis. These results suggest that the treatment with AR–ASR combinations during different infarct periods might be useful approach to heart protection.
